# The Formulation and Evaluation of Customized Prednisolone Gel Tablets Prepared by an Automated Extrusion-Based Material Deposition Method

**DOI:** 10.3390/pharmaceutics16121532

**Published:** 2024-11-29

**Authors:** Marina Tihhonova, Andres Meos, Sari Airaksinen, Jaan Aruväli, Niklas Sandler Topelius, Jyrki Heinämäki, Urve Paaver

**Affiliations:** 1 Faculty of Medicine, Institute of Pharmacy, University of Tartu, Nooruse 1, 50411 Tartu, Estonia; maratih@gmail.com (M.T.); andres.meos@ut.ee (A.M.); jyrki.heinamaki@ut.ee (J.H.); 2 CurifyLabs Oy, Salmisaarenaukio 1, 00180 Helsinki, Finland; sari.airaksinen@curifylabs.com (S.A.); niklas.sandler@curifylabs.com (N.S.T.); 3 Department of Geology, Institute of Ecology and Earth Sciences, University of Tartu, Ravila 14a, 50411 Tartu, Estonia; jaan.aruvali@ut.ee

**Keywords:** automated extrusion-based material deposition, gel tablet, prednisolone, solid-state change, dissolution in vitro, storage stability, veterinary medicine

## Abstract

**Background/Objectives**: An automated extrusion-based material deposition is a contemporary and rapid method for pharmaceutical dose-dispensing and preparing (printing) individualized solid dosage forms. The aim of this study was to investigate and gain knowledge of the feasibility of automated extrusion-based material deposition technology in preparing customized prednisolone (PRD)-loaded gel tablets for veterinary applications (primarily for dogs and cats). **Methods**: The PRD loads of the extrusion-based deposited gel tablets were 0.5% and 1.0%, and the target weights of tablets were 0.250 g, 0.500 g, and 1.000 g. The effects of the material deposition processes on the physical solid state, in vitro dissolution, and the physicochemical stability of PRD gel tablets were investigated. **Results**: The small-sized gel tablets presented a uniform round shape with an exceptionally smooth outer surface texture. The actual average weight of the tablets (n = 10) was very close to the target weight, showing the precision of the process. We found that PRD was in a pseudopolymorphic sesquihydrate form (instead of an initial PRD crystalline form II) in the gel tablets. In all the immediate-release gel tablets studied, more than 70% of the drug load was released within 30 min. The soft texture and dimensions of gel tablets affected the dissolution behaviour in vitro, suggesting the need for further development and standardization of a dissolution test method for such gel tablets. A short-term storage stability study revealed that the content of PRD did not decrease within 3 months. **Conclusions**: Automated extrusion-based material deposition is a feasible method for the rapid preparation of gel tablets intended for veterinary applications. In addition, the present technology and gel tablets could be used in pediatric and personalized medicine where precise dosing is crucial.

## 1. Introduction

Traditionally, pharmaceuticals are mass-produced in specific local industrial plants, and, therefore, final products, such as conventional tablets and capsules, have a limited number of alternative standardized doses. The levels of standardized doses are usually determined in early-phase clinical trials, which aim to achieve a safe therapeutic effect for most of the population [1]. However, a single standardized dose may not be suitable for all patients due to the varying characteristics of patients, such as gender, age, body mass, genetic profile, physiological properties, lifestyle choices, and individual preferences [2,3,4]. As a result, the demand for personalized medicines has motivated scientists to discover new methods that can be used for producing individualized dosages and formulations [5,6,7]. In the past, manual compounding was the cornerstone of preparing these specialized medicines in pharmacies and hospital pharmacies [8]. These compounded medicines or extemporaneously-prepared drug preparations still play vital roles in meeting unique patient needs today. Contemporary printing and material deposition technologies have the true potential to modernize traditional manual compounding of these patient-tailored drug preparations [2,4].

Pharmaceutical 3D printing with recent modifications hold great promises to over-come the well-known challenges related to traditional pharmaceuticals both in human and veterinary medicines. Using modern 3D printing technologies, active pharmaceutical ingredients (API) can be formulated into imaginative dosage forms that meet the specific needs of patients, and these products can be prepared on a small scale [9,10,11]. Semisolid extrusion (SSE) 3D printing technologies were successfully used to print medicines for patients in clinical trials [12,13,14]. Recently, Sjöholm et al. [15] investigated the use of three SSE 3D printers for the extemporaneous manufacturing of prednisolone (PRD) containing orodispersible films for veterinary use. To date, 3D printing technologies have been applied mainly for tissue engineering applications in veterinary medicine [16].

Automated extrusion-based material deposition (inspired by SSE 3D printing) is a promising contemporary printing technology, since it typically operates in low temperatures and allows for the use of traditional pharmaceutical-grade excipients [17]. The method was originally designed for dose-dispensing applications, but it also enables the rapid preparation (printing) of solid dosage forms, such as gel tablets, that have the desired (personalized) dose, design, dimensions, and surface texture. Therefore, the present material deposition method (like established 3D printing technologies) is applicable for preparing specialized solid oral dosage forms for different patient groups, such as polypills for elderly people, orodispersible drug preparations for patients with swallowing difficulties, and pet-friendly tablets for veterinary use. It is evident that such versatile and customized drug delivery systems will also improve patient adherence to drug treatments. Even though automated extrusion-based material depositions and 3D printing technologies have not yet been widely used in producing personalized medicines, their future uses in hospitals and pharmacies are evident [2,17,18,19,20].

Polymorphism is the ability of chemical compounds (such as APIs) to exist in multiple crystal forms with different arrangements of molecules within the unit cell. Solvates that contain water or organic solvents in their crystal structures are called pseudopolymorphs. Numerous APIs present a polymorphism or a pseudopolymorphism. The polymorphic forms of APIs have the same chemical composition but different conformational or orientational arrangements of molecules [21,22]. This leads to variations in the physicochemical properties of polymorphs, such as melting point, solubility, and chemical stability [23]. Changes in the polymorphic form or solvate in a crystal structure of API during manufacturing processes or storage can lead to alterations in drug properties and changes in the performance of the final dosage form. Therefore, controlling polymorphism in the manufacturing of pharmaceuticals is of the utmost importance to ensure the final quality, efficacy, and safety of medicinal products [24,25]. X-ray diffraction (XRD) and differential scanning calorimetry (DSC) are the methods of choice for investigating and controlling the physical solid-state changes in APIs.

Prednisolone (PRD) is a glucocorticoid widely used in the form of oral liquids, oral suspensions, conventional tablets, and oral disintegrating tablets for the drug treatment of many different inflammatory conditions [26]. Anhydrous PRD has two polymorphic forms, PRD I (monoclinic) and PRD II (orthorhombic), and one sesquihydrate form [27]. PRD I has a significantly higher melting temperature (236.5 °C–239.0 °C) compared to PRD II (223.8 °C–227.7 °C) (DSC) [27,28,29]. The melting point of PRD’s sesquihydrate form is between 238.3 °C and 238.8 °C (DSC). PRD II is thermodynamically the most stable at room temperature, and PRD I is the most stable polymorph above the transition point [27]. Subsequent studies confirmed that the two anhydrous polymorphic forms of PRD exhibit an enantiotropic relationship at normal pressure [29]. Bauer et al. [28] reported that the transition of PRD II to PRD I was not evident under the compression pressure range applied in industrial-scale tablet production or any other established pharmaceutical manufacturing process. Several studies have shown that the decomposition of PRD sesquihydrate leads to the formation of PRD I (metastable at an ambient room temperature), and the present transition of a sesquihydrate form occurs at 90 °C [22,27,29]. Recently, Lemercier and co-workers [22] found, perhaps surprisingly, the third (3rd) pseudopolymorphic form for PRD, which was isolated and identified as an isomorphous desolvate of the PRD sesquihydrate. However, the present PRD III form was highly metastable, which could be challenging from a pharmaceutical formulation development point of view [22].

The aim of this study was to investigate and gain knowledge of the feasibility of automated extrusion-based material deposition technology in preparing the customized small-sized gel tablets for veterinary applications (primarily for dogs and cats). The gel tablets are soft and elastic tablets with a pleasant mouth feel and texture, thus also enabling chewing of the drug preparations. Therefore, such small-sized “semisolid” tablets could be considered as applicable for veterinary oral drug therapy. The effects of extrusion-based material deposition on the physical solid state, in vitro dissolution and physico-chemical stability of PRD-loaded gel-tablets were investigated. Raman spectroscopy, Fourier transform infrared (FTIR) spectroscopy, and XRD were used for investigating the physical solid-state changes and other potential process-induced transformations (PITs) of PRD. The prepared gel tablets were also exposed to a short-term 3-month storage stability study in a refrigerator (+3–8 °C).

## 2. Materials and Methods

### 2.1. Materials

Micronized PRD powder, PRD #1 (Supplier: Thermo Fisher Scientific Inc., United Kingdom, Producer: China), with a purity of 99%, was used as a reference substance for all physicochemical analyses, except for the HPLC analysis. Another PRD powder, PRD #2 (Ph.Eur., Thermo Fisher Scientific Inc., United States), with a purity of 99%, was used as an API in the automated extrusion-based material deposition formulations and as a reference substance in the HPLC analysis.

For the HPLC analysis, acetonitrile (Honeywell/Riedel-de Haën™, Germany) and methanol (Sigma-Aldrich Chemie GmbH, Germany) were used as organic solvents in a mobile phase. For the dissolution tests, purified water (Millipore Milli-ro 12 Plus, Merck KGaA, Germany) was used as medium. The gel used for automated extrusion-based material deposition was Curavet™ (CurifyLabs Oy, Finland). The Curavet™ ink base consisted of microcrystalline cellulose (MCC), gelatine, and water (total 92% *w*/*w*), and additionally, small amounts (8% *w*/*w*) of extrusion-based material deposition supportive additives. The present ink base was developed primarily for veterinary applications. Curavet™ ink base has a simple composition, is feasible for extrusion-based deposition process (viscosity and consistency), and is safe for veterinary medicine applications (pets).

### 2.2. Methods

#### 2.2.1. Preparation of Gel Tablets

**Figure 1:**
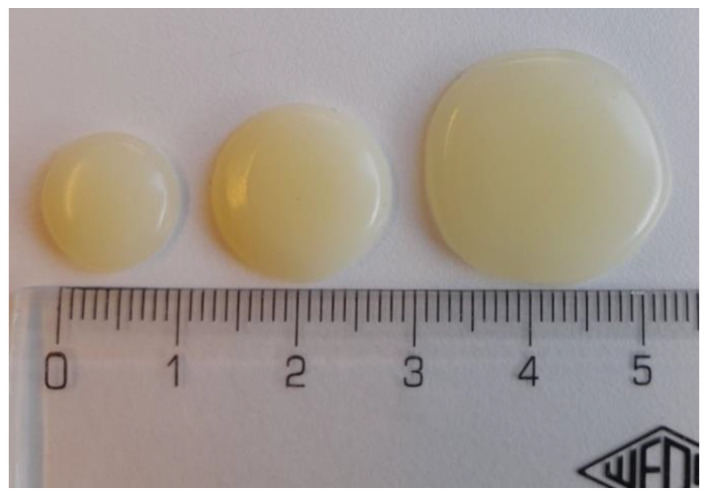
Photograph of the gel tablets with different target weights prepared by an automated extrusion-based material deposition method. From left to right: tablet 0.250 g, tablet 0.500 g, and 1.000 g mass.

The immediate-release gel tablets loaded with PRD were prepared by using a bench-top automated extrusion-based material deposition system (Dose Robot MiniLab, Finland). The material deposition (printing) temperature used was 42–45 °C and the printing rate was 16 gel tablets/minute. The ink base used was Curavet™ and the solvent was purified water. The concentrations (loads) of PRD in the gel tablets were 0.5% and 1.0%, and the target weights of the tablets were 0.250 g, 0.500 g, and 1.000 g (Figure 1). The gel tablets were directly packed and placed into a refrigerator (+3–8 °C) for further testing and the storage stability test. The tablets were individually packed in blisters (B) or Falcon centrifuge tubes (T). Three batches (I–III) of gel tablets were prepared (the batch I gel tablets; however, were used for the preliminary tests only). In addition, the corresponding gel tablets without API were used as reference tablets.

#### 2.2.2. Physicochemical Characterization of Gel Tablets

##### Scanning Electron Microscopy

The physical appearance and surface morphology of PRD crystals and gel tablets were imaged by means of scanning electron microscope (Zeiss EVO^®^ 15 MA, Zeiss, Germany). The samples were mounted on aluminum stubs with a conductive carbon film and were magnetron-sputter coated with a 3 nm platinum layer in an argon atmosphere before microscopy. The gel tablets were weighed with a Denver Instrument APX-200 analytical balance (Cole-Parmer Instrument Company LLC, USA), and the diameter and height were measured with an Ironside^®^ DIN862 calliper (Ironside International, France).

##### Vibrational Spectroscopy

Raman and FTIR spectroscopy methods are widely used in pharmaceutical material characterization, since they provide chemical structure analyses of APIs and excipients without any sample preparation. The solid-state properties and potential solid-state transformations of PRD were studied with a BWS415 i-Raman Miniature spectrometer (B&W TEK Inc., USA) (equipped with a CCD detector 2048 × 64, laser with a wavelength of 785 nm, and a power of 100 mW). For FTIR spectroscopy, IR Prestige-21 spectrometer (Schimadzu Corp., Japan) equipped with a Golden Gate ATR crystal, Specac Ltd. (UK), was used. With both vibrational spectroscopy methods, the spectra were collected from the surface of the gel tablets without any sample preparation.

##### X-Ray Powder Diffraction

X-ray powder diffraction (XRPD) is a powerful method for the solid-state analysis of pharmaceutical solids. The XRPD patterns of raw materials and gel tablets were obtained by using an X-ray diffractometer (D8 Advance, Bruker AXS GmbH, Germany). The XRPD experiments were carried out in a symmetrical reflection mode (Bragg–Brentano geometry) with CuK_α_ radiation (1.54 Å). The angular range was from 5° 2-theta to 35° 2-theta with steps of 0.2° 2-theta. The scattered intensities were measured with the LynxEye 1-dimensional detector with 165 channels. The operating voltage and current were 40 kV and 40 mA, respectively.

##### High-Performance Liquid Chromatography

The high-performance liquid chromatography (HPLC) setup used for the content analysis and in vitro dissolution of gel tablets consisted of a Shimadzu HPLC Prominence modular system (Shimadzu Europa GmBH, Germany), two high-pressure pumps LC-20AD, autosampler Nexera X2 SIL-30AC, photodiode detector Shimadzu SPD-M20A, and a column oven CTO-20AC. The system was controlled by LabSolutions version 5.3 (Shimadzu Corp., Japan). A Phenomenex Luna C18 (2) 250 × 4.6 mm (5 µm) column was used for the HPLC analysis. The composition of mobile phase was a 50:50 (V/V) mixture of (a) water and (b) 50:50 (V/V) mixture of acetonitrile and methanol (i.e., one litre of eluent contained 50% water, 25% acetonitrile, and 25% methanol). The method was isocratic, the column temperature was 40 °C, the flow rate was set as 1 mL/min, and the injection volume was 10 µL. UV absorption was detected at 254 nm (USP). The retention time for a PRD peak was approximately 8 min. For the content analysis, the gel tablets were dispersed in a water-acetonitrile 60:40 (V/V) mixture for 15 min (at about 30 °C) using an ultrasonic bath.

##### Dissolution Test In Vitro

The in vitro dissolution tests of gel tablets were conducted following the modified USP 35 paddle method (apparatus 2) intended for PRD conventional immediate-release tablets [30]. The dissolution tests were performed in a Sotax AT 7 Smart dissolution test apparatus (Sotax AG, Switzerland) equipped with an Ismatec IPC 8 ISM 931 peristaltic pump (Cole-Parmer Instrument Company LLC, USA) and Specord 200 Plus spectrophotometer (Analytik Jena GmbH, Germany). We also used an ultraviolet (UV) spectrophotometer Shimadzu UV-1800 (Schimadzu Corp., Japan) in the in vitro drug release studies of gel tablets. The analytical wavelength was 246 nm (USP). The rotating speed of the paddles was 50 rpm (in line with the USP method for conventional immediate-release tablets) and 100 rpm. The dissolution medium was purified water (900 mL) at 37 °C ± 0.5 °C. The samples were automatically withdrawn by using a peristaltic pump at specific time intervals (every 3 min) for UV spectrophotometer. For the HPLC analysis, the samples were taken manually at 7 min, 15 min, 30 min, and 60 min. The number of parallel gel tablets used in the dissolution tests was in most cases ten (n = 10), but in some dissolution tests, a smaller number of parallel gel tablets (n = 4 or 6) was used. For quantifying the PRD amount released, the dissolution test protocol also included one reference gel tablet without API in one test vessel and a freshly prepared PRD standard solution (5 mg/l000 mL) in another vessel as a reference solution. In the dissolution tests, the gel tablets were dispersed in a dissolution medium (purified water). Prior to the assay of PRD content, the samples were centrifuged for two minutes at 1500 g, and the clear supernatant was used for the HPLC analysis.

## 3. Results and Discussion

In the present study, the small-sized, soft and elastic gel tablets were developed and prepared for veterinary oral drug therapy (i.e., for cats and dogs). The gel tablets provide a pleasant mouth feel and texture, thus enabling also a chewing option for drug administration. To improve pet compliance, gel tablets can be flavoured with a meat or any other relevant taste. Moreover, such gel tablets and extrusion-based deposition technology could also find uses in human healthcare, e.g., in pediatric and personalized medicine, where precise dosing is crucial. Recently, Sandler Topelius et al. [17] investigated the performance of the extrusion-based deposition technology in preparing propranolol hydrochloride gel tablets specifically tailored for pediatric use. The authors succeeded in developing gel tablets that meet the pharmacopeial quality standards and address the unique needs and requirements of pediatric patients [17].

### 3.1. Physical Appearance and Surface Morphology

The gel tablets presented a uniform round shape with an exceptionally smooth outer surface texture (as shown in Figure 1). Table 1 summarizes the average weight, diameter, and height of the gel tablets. The actual weight of the gel tablets was very close to the target weight of the tablets, thus suggesting the feasibility of the automated extrusion-based material deposition process. The gel tablets were elastic and slightly bending, which makes the height measurements laborious and time-consuming.

**Figure 2:**
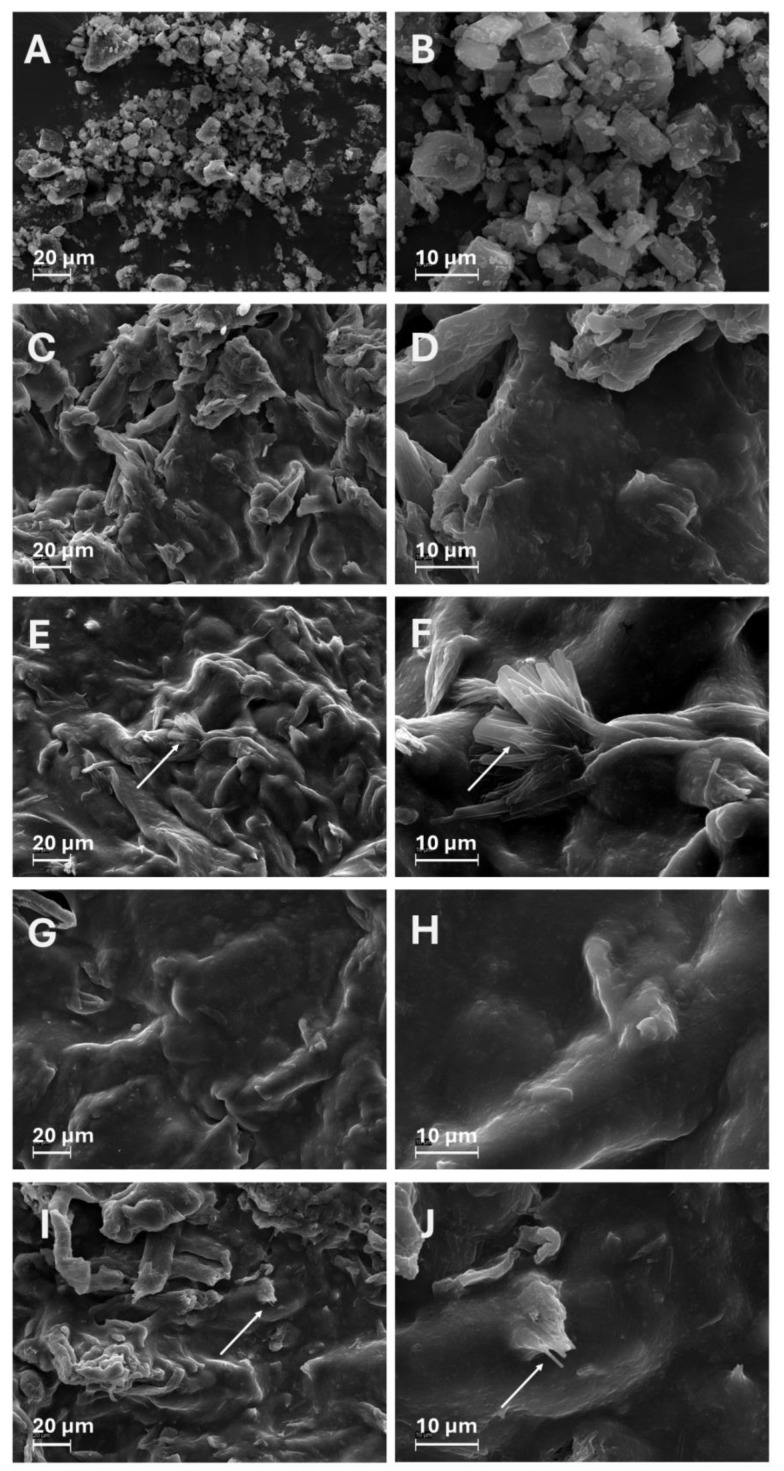
(**A**,**B**): Scanning electron microscopy (SEM) images of prednisolone (PRD) #1 powder crystals. (**C**–**F**): The surface of gel tablets. The reference gel tablet without API (**C**,**D**). The gel tablet (1.000 g) containing 10 mg (1%) of PRD (**E**,**F**). (**G**–**J**): The cross-section of gel tablets. The reference gel tablet without API (**G**,**H**). The gel tablet (1.0 g) containing 10 mg (1%) of PRD (**I**,**J**). Partial re-crystallization of PRD on the surface of gel tablet (**E**,**F**) and to a minor extent inside a gel tablet (**I**,**J**) is indicated with white arrows. Magnification in the left column 1500× (scale bar 20 µm) and in the right column 5000× (scale bar 10 µm).

Figure 2 shows the SEM images on PRD #1 powder particles and gel tablets. PRD #1 powder was composed of crystalline particles with a micronized particle size (less than 20 µm) and an irregular (or partially cubic/square-like) shape (Figure 2A,B). The particle (crystal) shape of a micronized PRD #1 powder (shown in Figure 2) differed from the crystal shape of the known polymorphic forms of PRD described in the literature [22,27,28,29]. The known polymorphic forms of PRD present typically elongated crystals, thus significantly differing from the crystal shape of PRD #1 powder. The present difference is obviously due to the micronization process used in preparing a PRD #1 powder. The SEM micrographs on the surface of gel tablets are shown in Figure 2C–F. As shown in Figure 2F (the SEM micrograph with higher magnification of 5000×), the elongated PRD crystals can be seen on the surface of PRD gel tablets.

This suggests partial re-crystallization of PRD on the surface of the gel tablet after automated extrusion-based material deposition. Such crystals are not detectable on the surface of the reference gel tablets (the composition without API) (Figure 2D). The SEM micrographs on the cross-section of gel tablets are shown in Figure 2G–J. In these SEM micrographs, the PRD crystals (or crystal clusters) are not distinguishable, thus suggesting the homogeneous consistency of gel tablets. However, the inclusion of API in the gel tablets resulted in some conformational changes (Figure 2I,J) in the internal structure of the tablets in comparison to the reference gel tablets without API (Figure 2G,H).

### 3.2. Physical Solid-State Changes

PRD has two polymorphic forms, PRD I (monoclinic) and PRD II (orthorhombic), and the API also exhibits two solvate forms, PRD anhydrate and sesquihydrate [27,31]. PRD II is thermodynamically the most stable at room temperature and PRD I is the stable polymorph above the transition point [27]. The transition of PRD II to PRD I; however, is unlikely under the tablet compression or any other established pharmaceutical manufacturing process [28]. PRD has also a highly metastable pseudopolymorphic form, PRD III, which was isolated and identified as an isomorphous desolvate of the PRD sesquihydrate [22]. To date, no studies have been published on the physical solid-state changes associated with the semisolid extrusion-based deposition (printing) of prednisolone gel tablets.

**Figure 3:**
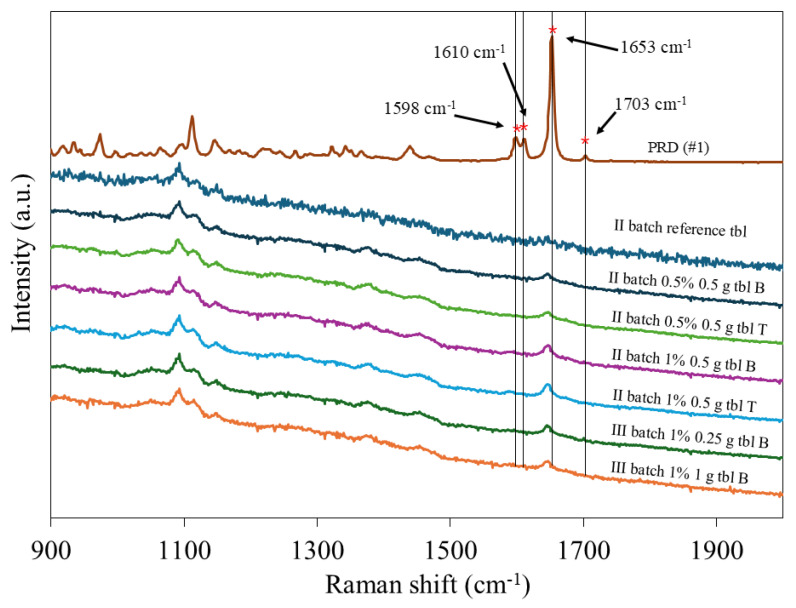
Raman spectra of prednisolone (PRD) #1 powder and gel tablets. Vertical lines and asterisks are used to indicate the most important peaks of PRD #1 powder. The Raman spectra of gel tablets are presented for both blister packaging (B) and tube (T) samples. * indicates characteristic peaks of PRD.

The Raman spectra of PRD #1 powder and gel tablets are shown in Figure 3. It is evident that the PRD #1 powder used in our study exists in polymorphic form II. The dissolution rate of PRD II is higher than that of PRD I in distilled water at 37 °C [32]. The Raman spectrum of PRD #1 shows two distinct peaks at the Raman shift of 1600 cm^−1^, thus indicating the presence of PRD polymorphic form II (Figure 3). However, the dominant Raman spectroscopy peak of PRD was not able to be clearly distinguished in the corresponding spectra of gel tablets most likely due to the low content of API (10 mg/1%). Moreover, the Raman spectroscopy peak of PRD was located at an average of 1645 cm^−1^ Raman shift, whereas the literature reveals that the characteristic peak of PRD is at a shift of 1653 cm^−1^ [33,34]. Due to the low concentration of PRD, the other characteristic peaks of PRD were barely visible in the Raman spectra of gel tablets as well.

**Figure 4:**
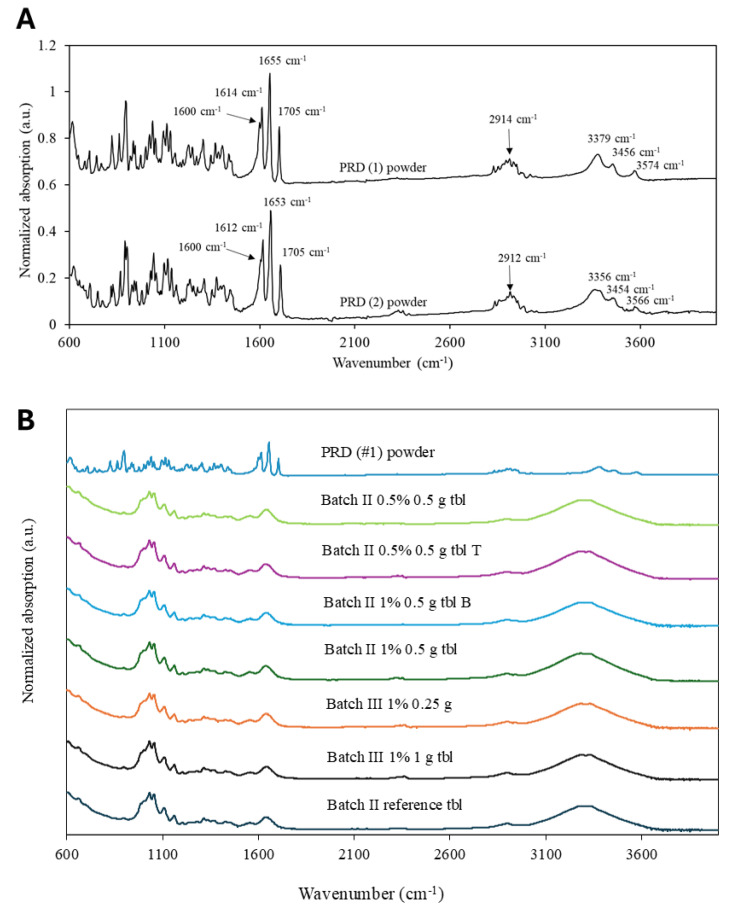
(**A**) Fourier-transform infrared (FTIR) spectra of prednisolone (PRD) #1 and PRD #2 powders. (**B**) FTIR spectra of gel tablets and PRD) #1 powder investigated in the present study. The FTIR spectra of gel tablets are presented for the tablets packed in a blister pack (B) and in a tube (T).

Figure 4 shows the Fourier-transformed infrared spectroscopy (FTIR) spectra for both PRD powders (Figure 4A) and gel tablets (Figure 4B). The FTIR spectra of PRD #1 and #2 powders (shown in Figure 4A) provide evidence that both PRD powders exist in a polymorphic II form. This is confirmed by the presence of FTIR spectroscopy peaks for both PRD powders at wave numbers 1705 cm^−1^, 1653–1655 cm^−1^, 1612–1614 cm^−1^, and 1600 cm^−1^, which correspond with the characteristic peaks for a PRD polymorphic II form according to the published literature [33]. Perhaps surprisingly, we did not find any significant differences between the FTIR spectra of the reference gel tablets and the PRD-loaded gel tablets (Figure 4B). This was likely due to the amount of PRD used in the gel tablets, which was perhaps too small for the determination of the exact solid-state form of the API by means of FTIR spectroscopy. Moreover, it is obvious that the excipients used in gel tablets masked the display of the peaks. Recently, Sjöholm et al. [15] reported that the broad peak of carrier polymer (hydroxypropyl cellulose, HPC) at 3600–3100 cm^−1^ hides the characteristic peaks of PRD in the SSE 3D-printed drug-loaded orodispersible films.

**Figure 5:**
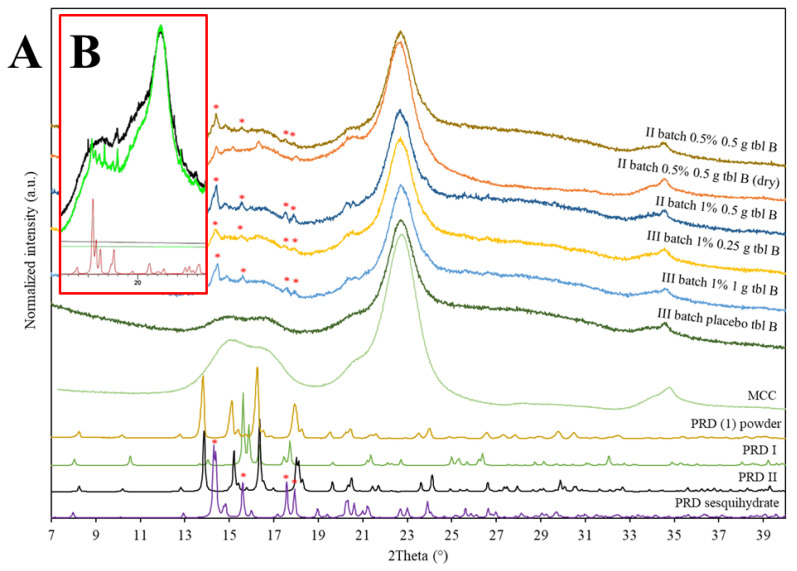
X-ray diffraction (XRD) patterns for (**A**) prednisolone (PRD) #1 powder and gel tablets, and for (**B**) the physical mixtures of Curavet™ components with (green line) and without (black line) PRD. The target weight for the gel tablets were 0.250 g, 0.500 g, and 1.000 g, and the tablets were stored in blister packs. Key: MCC = microcrystalline cellulose, PRD I = PRD polymorphic form I, PRD II = PRD polymorphic form II, and * = the major peaks of a PRD sesquihydrate form. The corresponding theoretical X-ray diffractograms are retrieved from the Cambridge Structural Database [35].

The XRD analyses were conducted immediately after the extrusion-based material deposition process and after the storage of gel tablets in a refrigerator (+3–8 °C) for approximately 3 months. Figure 5 presents the XRD patterns for PRD #1 powder and the gel tablets (Batches II and III). The XRD results revealed that PRD is in a sesquihydrate form in gel tablets indicated with asterisks. It is evident that as the gel tablets were partially dehydrated, and the pseudopolymorphic sesquihydrate form of PRD transformed into a polymorphic II form. However, due to the minimal peaks of XRD for the gel tablets with a drug content of 1.0%, this observation would need a further verification.

For comparison, three theoretical X-ray diffractograms retrieved from the Cambridge Structural Database (CSD) are included in Figure 5. The structure reference codes used for generating the polymorphic I, II and sesquihydrate forms of PRD were JIWPEL01, JIWPEL and JIWPIP, respectively [35]. Based on the comparison between the theoretical and experimental diffractograms of PRD powder, it can be concluded that PRD #1 presents a crystalline structure characteristic of a PRD II polymorph. As seen in Figure 5, the XRD pattern for a PRD #1 powder corresponds well with the diffractogram of the polymorphic II form of PRD retrieved from a CSD [35].

The XRD patterns for the gel tablets were mainly governed with the characteristic peak of MCC (the main excipient component present in a Curavet™ base). The X-ray diffractogram of the reference gel tablets (without any API) (Batch II) displayed an “amorphous halo” characteristic to amorphous materials (i.e., the two main excipients of a Curavet™ base). We observed that under the XRD analysis, some of the gel tablets were dehydrated. When comparing the XRD patterns of dehydrated (“dry”) gel tablets and non-dehydrated gel tablets (Figure 5), significant changes in the corresponding patterns can be observed.

The non-dehydrated gel tablets showed the diffraction peaks characteristic to the sesquihydrate form of PRD, most likely due to the incorporation of water into the crystal lattice of API. As seen in Figure 5, with the non-dehydrated gel tablets the position of the XRD peaks (PRD) have shifted, thus indicating the transformation of the sesquihydrate form of PRD to a polymorphic II form.

We also investigated the effect of the short-term storage of gel tablets on the physical solid state of API. The XRD results revealed that the storage of gel tablets in a refrigerator (+3–8 °C) for approximately 3 months did not lead to the physical solid-state changes in API in the tablets. Figure 5 shows that the XRD pattern for the non-dehydrated gel tablets of PRD showed the characteristic peaks of a PRD sesquihydrate form. Moreover, the HPLC analysis revealed that the content of PRD in the d gel tablets remains stable during a 3-month storage period. The API content determined by means of HPLC for the “fresh” gel tablets was 103.3 ± 0.8% (n = 9), and for the “aged” gel tablets, 102.7 ± 2.6% (n = 7).

### 3.3. Dissolution In Vitro

According to the literature, a wide range of excipients has been used in the commercial immediate-release PRD tablets, and the excipients present in these drug products do not have a significant effect on the dissolution and oral bioavailability of PRD, and hence no impact on its clinical use [31]. The current data on solubility, oral absorption, and permeability strongly suggest a BCS Class 1 classification for PRD [31].

The in vitro dissolution tests were conducted following the USP 35 monograph paddle method (apparatus 2) stated for the conventional immediate-release tablets of PRD [30]. We found that only an average of 50 ± 1.4% of the nominal amount of API was released from gel tablets after 30 min (HPLC) in the dissolution test using a paddle speed of 50 rpm. Therefore, the gel tablets did not comply with the requirements specified in the USP 35 monograph (not less than 70%/30 min) [30].

**Figure 6:**
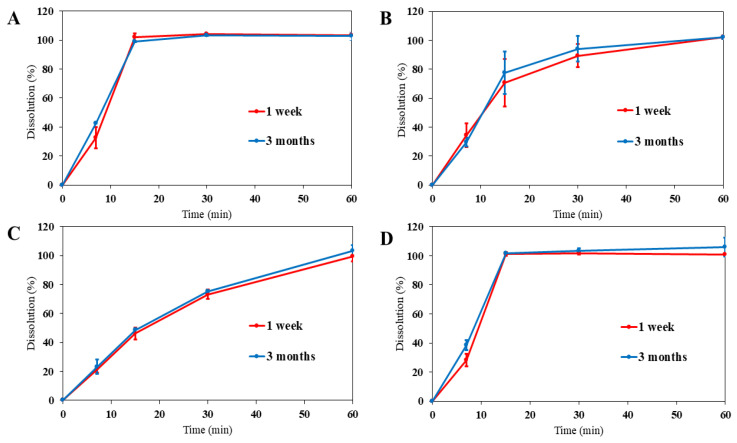
In vitro dissolution profiles of prednisolone (PRD) gel tablets (n = 4–10). The dissolution tests were conducted one week after the gel-printing process and after the storage of three months in a refrigerator (+3–8 °C). Key: (**A**) PRD gel tablets 0.5%/0.500 g originally packed in blisters (Batch II); (**B**) PRD gel tablets 1%/0.500 g packed in blisters (Batch II); (**C**) PRD gel tablets 1%/0.250 g packed in blisters (Batch III); (**D**) PRD gel tablets 1%/1.000 g packed in blisters (Batch III). The standard deviation (SD) of all dissolution results was less than 16.3%, and in most cases, in the range of 0.5–3.5%.

The in vitro dissolution tests for the gel tablets were also conducted using a paddle rotating speed of 100 rpm. A total of twelve (12) in vitro dissolution tests were performed involving two tests for each type of gel tablets, and the results are summarized in Figure 6. The dissolution tests were conducted one week after the automated SSE material deposition process and after three months of storage in a refrigerator (+3–8 °C). Figure 6A shows the in vitro drug release of the gel tablets (0.5 g) with the PRD content of 2.5 mg (0.5%). The gel tablets disintegrated within 15 min (observed by a visual inspection). The in vitro dissolution results revealed that the amount of drug released at 15 min was 101.9 ± 2.5% (for the tablets kept in a blister pack for one week).

We also found that the gel tablets (0.500 g) with the PRD content of 2.5 mg (0.5%) exhibited a distinctive behaviour in the dissolution test compared to the other gel tablets studied. Instead of sinking to the bottom of the test vessel like their counterparts, the gel tablets (originally packed in blisters or tubes) remained floating in the vessel. The disintegration of the gel tablets was driven by the centrifugal force caused by stirring. Notably, the most significant deviations in the release of API were observed specifically with these floating tablets. Such large variation in the dissolution of gel tablets could be due to the absence of degassing for the gel utilized for automated extrusion-based material deposition. The gel could have contained air bubbles, thus preventing the gel tablets from descending to the bottom of the test vessel.

Figure 6B presents the in vitro dissolution profiles for the PRD gel tablets (0.500 g) with the PRD content of 5.0 mg (1.0%) packed in blisters (Batch II). With these gel tablets, 70.5 ± 16.3% of the drug load was released within 15 min (the dissolution test one week after the printing process). As shown in Figure 6B, the entire drug load was completely released within approximately 60 min. The PRD gel tablets (0.500 g) with the PRD content of 5.0 mg (1.0%) exhibited clearly slower drug release compared to that observed with the corresponding gel tablets with a lower API load (2.5 mg). Furthermore, the variation in the dissolution of individual gel tablets (0.500 g) with the PRD content of 5.0 mg (1.0%) was large, thus suggesting that the gel tablets disintegrated unevenly. Unlike the floating PRD gel tablets (0.500 g) with a lower API load (2.5 mg), the present higher API-loaded gel tablets (1%/0.500 g) showed a tendency to settle down to the bottom of the test vessel.

Figure 6C shows the dissolution curves of gel tablets (0.250 g) with the PRD content of 2.5 mg (1.0%) packed in blisters. Completely different release kinetics were observed with the present gel tablets, since the tablets sank to the bottom of the test vessel and were “trapped” in there (the tablets were swirled due to the movement of a dissolution medium). The drug release of these gel tablets was the slowest, and only 73.2 ± 3.3% of the API load was released within 30 min. As seen in Figure 6C, approximately 100% of the drug load (99.5 ± 3.4%) was released within 60 min from the gel tablets. The slow drug release in vitro could be explained by the smallest surface area of these PRD gel tablets (0.250 g) with the PRD content of 2.5 mg (1.0%) and/or the adhesion of the tablets to the bottom of the dissolution vessel.

Figure 6D shows the dissolution profiles of PRD gel tablets (1.000 g) with the PRD content of 10 mg (1.0%) packed in blisters (Batch III). The drug release from such gel tablets was very fast, and 100% (101.3 ± 1.2%) of the drug load was released within approximately 15 min. The dissolution profile of these gel tablets was close to that observed with the PRD gel tablets (0.500 g) with the PRD content of 2.5 mg (0.5%) (Figure 6A).

**Figure 7:**
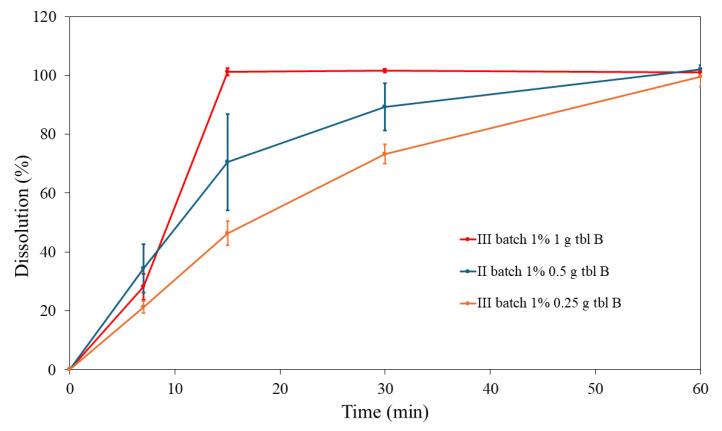
In vitro dissolution profiles of prednisolone (PRD) gel tablets with a drug load of 1% (n = 4–10). The weight of the tablets were 0.250 g (Batch III), 0.500 g (Batch II), and 1.000 g (Batch III). The dissolution tests were conducted one week after the printing process. The gel tablets were packed in a blister pack. The standard deviation (SD) of dissolution results was less than 5% for the gel tablets 0.25 g and 1.0 g, and less than 16.3% for the gel tablets 0.5 g.

We also compared the dissolution profiles of PRD gel tablets with the highest drug load of 1.0% having a different tablet weight (0.250 g, 0.500 g, and 1.000 g) and diameters (approx. 1.1 cm, 1.5 cm, and 2.0 cm) (as shown in Table 1 and Figure 1). The effects of these two parameters on the dissolution behaviour of gel tablets are shown in Figure 7. The outer surface area of the present PRD (1%) gel tablets is directly related to the weight of the tablets: the tablets with an average weight of 1.000 g present the highest outer surface area, while the tablets with an average weight of 0.250 g have the smallest outer surface area. As seen in Figure 7, the drug release was very much dependent on the weight and surface area of the PRD (1%) gel tablets: the higher the outer surface area (and the larger the tablet weight), the faster the drug release of PRD in vitro.

Figure 7 shows that the drug release was quite uniform with the PRD (1%) gel tablets up to 7 min, and PRD gel tablets (1%/0.500 g) exhibited the fastest drug release at this time point. After this time point, the drug release was clearly the fastest with PRD gel tablets (1%/1.000 g), and with these tablets, the drug load was completely released (101.3 ± 1.2%) within 15 min. In contrast, the PRD gel tablets (1%/0.250 g) clearly exhibited the slowest drug release (only 46.3 ± 4.1% of a PRD load released within 15 min). With these low-dose tablets; however, 100% of the drug load was completely released within 60 min. With the PRD gel tablets (1%/0.500 g), approximately 70% (70.5 ± 16.3%) of the drug load was released at 15 min, and the drug release was complete within 60 min.

The present results are in accordance with the in vitro dissolution results obtained with the SSE 3D-printed orodispersible films loaded with PRD which rapidly dissolved and reached 80% drug release within 50-60 min in purified water at 37 °C [15]. When dosing, such gel tablets to animals, the drug release is most likely faster as the animal could chew the tablets upon administration.

### 3.4. Study Limitations

The limitations of this study include small sample size and short duration of stability testing. The small content of API in the gel tablets limited the interpretation of our Raman and FTIR spectroscopy results. The gel tablets were loaded with PRD and the generalization of the findings to the corresponding formulations of other APIs is not possible. In addition, we did not perform any animal studies to verify the applicability of the present gel tablets in veterinary drug therapy.

## 4. Conclusions

Automated extrusion-based material deposition (inspired by SSE 3D printing) is a contemporary and rapid method for preparing customized pharmaceutical gel tablets. In the present study, we developed and prepared oral PRD gel tablets for veterinary applications. The elastic gel tablets showed a uniform round shape with an exceptionally smooth outer surface texture. The actual weight of the gel tablets was very close to the theoretical target weight set, thus showing the feasibility of the process. PRD existed as polymorphic form II (XRD) in a powder form, and as a pseudopolymorphic sesquihydrate form (XRD) in the gel tablets. This suggests a PIT associated with the automated extrusion-based material deposition of the present API. It is also evident that the drying of gel tablets leads to the transformation of the PRD pseudopolymorphic sesquihydrate form back into the more stable polymorphic form II. However, this finding needs further verification. The soft texture and dimensions of gel tablets have an impact on the dissolution behaviour in vitro, suggesting the need for further development and standardization of dissolution test methods for such gel tablets. The short-term storage stability results reveal that the physical solid-state form and content of PRD in the gel tablets remain stable for at least 3 months when the gel tablets are stored in a refrigerator (+3–8 °C). The overall findings of this study emphasize the importance of accurately identifying the solid-state form of PRD and potential PITs in the gel tablets and highlight the need to adopt a suitable in vitro dissolution test method and conditions for such novel gel tablets. In conclusion, the present extrusion-based deposition technology and gel tablets could find uses in veterinary medicine and also in human healthcare, e.g., in pediatric and personalized medicine where precise dosing is crucial.

## Figures and Tables

**Table 1:** The weight and dimensions of prednisolone (PRD) gel tablets (n = 10) prepared by an automated extrusion-based material deposition method.

Gel Tablet Formulation	Target Weight (g)	Weight (g) (Mean ± SD)	Diameter (mm) (Mean ± SD)	Height (mm) (Mean ± SD)
#1	1.000	0.959 ± 0.015	20.45 ± 0.58	2.90 ± 0.14
#2	0.500	0.472 ± 0.006	15.08 ± 0.33	2.69 ± 0.17
#3	0.250	0.249 ± 0.006	10.94 ± 0.37	2.13 ± 0.06

## Data Availability

The original contributions presented in the study are included in the article and further inquiries can be directed to the corresponding author.
